# How should a cluster randomized trial be analyzed?

**DOI:** 10.1016/j.jeph.2024.202196

**Published:** 2024-02-10

**Authors:** Laurent Billot, Andrew Copas, Clemence Leyrat, Andrew Forbes, Elizabeth L. Turner

**Affiliations:** a The George Institute for Global Health, University of New South Wales, Sydney, Australia; b MRC Clinical Trials Unit at University College London, London, UK; c Department of Medical Statistics, London School of Hygiene and Tropical Medicine, London, UK; d School of Public Health and Preventive Medicine, Monash University, Victoria, Australia; e Department of Biostatistics and Bioinformatics and Duke Global Health Institute, Duke University, Durham, NC, USA

**Keywords:** Cluster randomized trials, Stepped-wedge trials, Cluster crossover trials, Analysis

## Abstract

In cluster randomized trials, individuals from the same cluster tend to have more similar outcomes than individuals from different clusters. This correlation must be taken into account in the analysis of every cluster trial to avoid incorrect inferences. In this paper, we describe the principles guiding the analysis of cluster trials including how to correctly account for intra-cluster correlations as well as how to analyze more advanced designs such as stepped-wedge and cluster cross-over trials. We then describe how to handle specific issues such as small sample sizes and missing data.

## Background

Cluster randomized trials (CRTs) are trials in which groups, or clusters, of individuals are randomized to one of two or more conditions [1]. Despite their expected loss in statistical power or, equivalently, their smaller effective sample size as compared to an individually randomized trial with the same number of participants [2], a CRT design is appropriate when the intervention under evaluation is at the cluster level or where there would be a risk of intervention contamination if individual-level randomisation were used [3]. As with standard individually-randomized trials, there are variations on the CRT design, such as the stepped-wedge (SW) CRT and the related cluster-crossover design. For more information on different designs, see [4].

When analyzing any type of CRT, it is essential to account for the expected correlation of outcomes within the same cluster and to recognize other design features in the analysis (e.g. the potential for confounding by time in the stepped-wedge design). Statistical methods that account for clustering of outcomes include cluster-level summary methods or individual-level regression methods, such as generalized linear mixed models and generalized estimating equations. In this article, we describe overall principles that must be followed in the analysis of CRTs, SW-CRTs and cluster-crossover designs. We then explain how to handle special issues such as missing data and how to appropriately analyze CRTs with a small number of clusters. Finally, we discuss how to analyze cohort designs [4] (i.e. multiple-period designs where the same participants are followed throughout) as well as how to accommodate more complex outcome correlation structures.

## Overall principles

### Intra-cluster correlation (ICC)

Outcomes among individuals from within the same cluster are expected to be correlated whereas outcomes among individuals from different clusters are independent. This phenomenon, called “intra-cluster correlation”, leads to an inflation of the variance and a loss of statistical power compared with an individually-randomized trial with the same total sample size. To produce correct inference and avoid results that are too liberal, statistical analyses must account for this correlation.

### Cluster-level analysis

To account for clustering, one can either perform an analysis at the cluster-level or at the individual-level. A cluster-level analysis is a two-step process where the first step consists in aggregating the outcome of interest at the cluster level. For example, if individual outcomes are of a binary (yes vs no) nature, the first step would consist in calculating the proportion of that outcome within each cluster. The second step would consist in comparing these cluster-level proportions using an appropriate statistical test such as a T-test. Different sets of weights can be applied to reflect unequal cluster sizes or unequal variances [5]. A major advantage of the cluster-level approach is that the within-cluster correlation does not need to be modeled, thus making the analytical process simpler. Its main disadvantage is its lack of flexibility, in particular if one wishes to perform covariate adjustments or examine interactions with individual-level covariates. Methods to adjust for individual covariates in cluster-level analyses involve two steps. The first step consists in running a regression on individual outcomes adjusting for individual covariates but without adjusting for clustering and without including the intervention effect. The second step consists in performing a cluster-level analysis of the residuals obtained from the individual-level regression [5]. Recent software has made this implementation simpler [6]. An alternative is to use an individual-level analysis while adjusting for clustering.

### Individual-level analysis

Due to its flexibility, the individual-level analysis is the most commonly used approach. It is typically conducted using either generalized linear mixed models (GLMM) or generalized estimating equations (GEE, [7]). The choice between the two methods is mainly driven by whether we wish to estimate a ‘cluster-specific’ or ‘population-average’ effect of the intervention. To adjust for the effect of clustering, the GLMM approach typically includes a random cluster effect. This implies that the resulting estimates are ‘cluster-specific’ or ‘conditioned’ on the cluster effect. Conversely, in the GEE approach, a working within-cluster correlation structure is used but the analysis model does not condition on the effect of the cluster and so is ‘marginal’ or ‘population averaged’. These two approaches are aligned for linear and logarithmic link models effect (i.e., when estimating difference between means, risk differences or risk ratios) but differ for the logistic link (estimating an odds ratio for the intervention effect). The GLMM approach can however be adapted to provide a population average intervention effect [ref]. One advantage of GEEs is that they can be used when there is uncertainty about the correlation structure because valid (‘robust’) standard error estimates can be calculated even when the correlation structure is misspecified, provided the marginal analysis model is correctly specified. In this paper, we consider both conditional models estimated using GLMM and marginal models estimated using GEE. We introduce the following basic linear mixed model for continuous outcomes where *Y*_ij_ is the outcome of individual i (*i* =1 ... m_j_) from cluster j (*j* = 1 to k), *β*_1_ is the fixed effect of the intervention, *u*_j_ is a normally-distributed random effect of the cluster and *ε*_ij_ is a normally-distributed individual-level error (with *u*_j_ and *ε*_ij_ assumed to be independent).

(Model 1) $$Y_{ij}=\beta_{0}+\beta_{1}\times \;\text{Intervention}\;+u_{j}+u_{ij}$$

The GEE analog of Model (1) excludes the random cluster effect but assumes that individual-level errors for individuals from the same cluster are correlated assuming a constant (‘exchangeable’) correlation. Both models can be generalized to accommodate other types of outcomes, for example to a logistic regression for a binary outcome and can be further adjusted for baseline covariates. If an exchangeable correlation is not realistic then additional random effects can be included in the GLMM. The working correlation for the GEE can also be adapted in principle, though in most statistical software the choice of possible correlation structures is limited.

A noteworthy extension of the parallel cluster trial is to add an observational baseline period to obtain additional outcome measurements [4] and increase power. Hooper et al. [8] recommend using either an analysis of covariance or a constrained baseline analysis, the latter extending Model 1 to a longitudinal model including outcomes collected during both the baseline and follow-up periods.

Example: The UKGRIS trial [9] was a cluster randomized trial of a risk stratification strategy using the Grace Risk Score in patients with suspected acute coronary syndrome. 38 hospitals (clusters) were randomized 1:1 to patient management by standard care or according to the risk stratification approach. The primary outcome (binary) was use of guideline-recommended management. A total of 22,473 participants were deemed eligible and included in the primary analysis. The effect of the intervention was estimated with GLMM using a logistic regression with a random cluster intercept. Fixed effects included the randomized intervention as well as stratification variables. The uptake of guideline-recommended processes was 77.3 % for the risk-stratification group and 75.3 % for standard care leading to a non-significant difference (odds ratio 1.16, 95 % confidence interval 0.70 to 1.92, *P* = 0.56). A naive analysis without adjusting for clustering, e.g. using a simple chi-square test, would have incorrectly produced a strongly significant result with a p-value < 0.001.

### Analysis of stepped-wedge designs

**Fig. 1:**
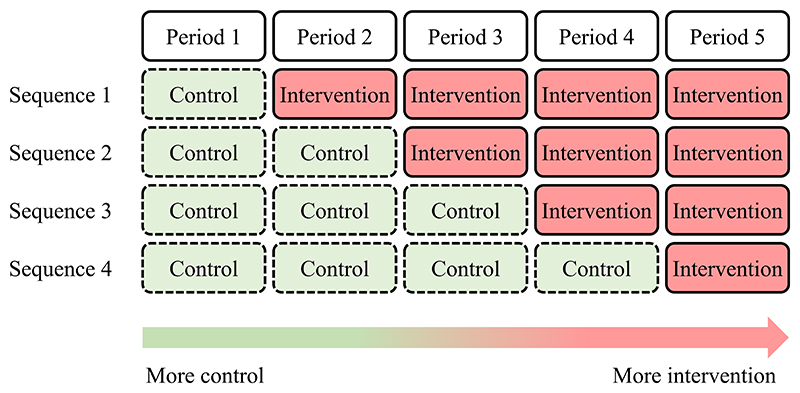
Example of stepped-wedge trial with 4 sequences (5 periods). In a stepped-wedge trial, more clusters are receiving the intervention in later periods, necessitating appropriate adjustment for time to avoid potential confounding biases.

The analysis of the stepped-wedge (SW) design follows similar principles to the analysis of the parallel-arm cluster design, but there are key differences. First, because the proportion of individuals exposed to the intervention increases over time in the SW design, there is inherent confounding between the effect of the intervention and the effect of time which can induce bias. This can happen for example when the incidence of an outcome varies by season. To avoid such potential biases, it is crucial to model the effect of time when analyzing a SW trial. This is typically done by adding a fixed categorical time effect to *Model 1*, that corresponds to the periods of the design. For example, in a standard stepped-wedge trial with four sequences (Fig. 1), the time effect will include 5 categories. It is also possible to model time with continuous terms, such as splines, when outcome data are collected in a continuous manner.

We should also consider whether, and if so how, the effect of the intervention may vary with time from its introduction. Interventions that are based on simple technologies, e.g. an ‘app’ to manage patients, may be expected to have an immediate and sustained effect. Other interventions may involve staff training that could be forgotten if not repeated, or conversely improve with experience e.g. surgical procedures.

The simplest model assuming that different participants are measured in each period (see “Special considerations - Analysis of cohort trials” section) and an immediate and sustained intervention effect, involves adding only the effect of the period to the model for a parallel-arm trial as follows:

(Model 2) $$Y_{ij}=\beta_{0}+\beta_{1}\times \;\text{Intervention}\;+\beta_{T}\times \;\text{Period}\;+u_{j}+\varepsilon_{ij}$$

Model (2) assumes a common time trend across all clusters, following the model introduced by Hussey and Hughes [10]. Consequently, the correlation between measurements on individuals is assumed to be the same for all individuals from the same cluster, whether they were recruited within the same period or a different period, i.e. data are assumed to be exchangeable. However, it is plausible that outcomes from individuals recruited from different periods are less correlated than outcomes from individuals recruited within the same period. This might be especially true when one period corresponds to the control and another to the intervention. Extensions to *Model 2* can be implemented to allow the effect of time or treatment to vary across clusters [11]. For example, the addition of a random cluster-by-period interaction term to the GLMM specifies a ‘block exchangeable’ correlation structure. This leads to two distinct correlation parameters: one for individuals from the same cluster and same period and another for individuals from the same cluster but from different periods. Other extensions include ‘auto-regressive’ or ‘decaying’ correlation structures [12]. When using GEE, most software does not allow the user the flexibility to choose structures such as block exchangeable; however, the GEE analysis remains robust to misspecification in the correlation structure.

When the effect of the intervention varies with time, an analysis using the basic model may not provide a meaningful ‘average’ effect; hence some authors have proposed extended models that allow the intervention effect to change with time since its introduction [13].

Example: The Devon Active Villages Evaluation (DAVE) trial was a SW cluster randomized trial of a community-level physical activity intervention [14]. 128 villages (clusters) were randomized to one of four sequences (Fig. 1) and outcomes were collected within each period from all villages using a postal survey. The primary outcome (binary) was reporting sufficient physical activity to meet internationally recognized guidelines. A random sample of residents in each village was taken to receive the survey, with a new sample taken for each period. To estimate the intervention effect, a logistic regression model was fitted by GEE with exchangeable working correlation. Although some decay in correlation between time periods might be realistic in this setting the GEE remains robust to misspecification of the correlation. Time period was included in the model (five categories), and the intervention indicator was determined by the intended exposure in the sequence (intention-to-treat). Post-hoc analyses assessed whether the intervention had either a delayed effect or an immediate effect that subsided.

### Analysis of cluster crossover designs

Cluster crossover (CRXO) trials share similarities with SW trials. Here clusters are also randomized to a sequence of intervention; however, unlike stepped-wedge trials where clusters can only transition from control to intervention, in a crossover trial the transition can be in either direction. This design is only validly used when it is possible to remove or “switch off” the intervention applied in the first period of the trial so that each cluster returns to its initial pre-trial state. The most common CRXO consists of two possible sequences, one from control to intervention, the other from intervention to control - also called the “AB/BA” design. Extensions to this basic design include more than two periods, for example with clusters alternating between control and intervention periods multiple times, or more than two possible sequences.

Assuming again a continuous outcome (Y_ijk_) and a GLMM, the basic model to analyze a cluster crossover trial consists of the following:

(Model 3) $$Y_{ijk\;}=\beta_{0}+\beta_{1}\times \;\text{Intervention}\;+\beta_{T}\times \;\text{Period}\;+u_{j}+t_{jk}+\varepsilon_{ij}$$

In Model (3), *t*_jk_ represents a random cluster-by-period interaction. The addition of this random effect implies that the within-cluster correlation is different for participants from the same cluster enrolled in the same period than for participants from the same cluster enrolled in two different periods.

The above model is also applicable to CRXO designs with more than two periods; however, as with the analysis of SW trials, more complex correlation patterns are possible [13]. It is important to note that the analysis of cluster trials using the above model relies on the assumption that there is no carry over effect, that is, the effect of one intervention does not persist into the next period. Carry over effects are generally addressed at the design stage, by either assuming they are implausible or implementing an appropriate washout period [4].

Example: The PEPTIC trial [15] was a pragmatic unblinded cluster crossover trial comparing two stress ulcer prophylaxis strategies (proton pump inhibitors (PPIs) vs histamine-2 receptor blockers (H2RBs)) in mechanically ventilated patients in 50 intensive care units (ICU). Each ICU was randomised to receive either PPI then H2RB or vice versa for two sequential 6-month periods. The primary outcome was in-hospital mortality. The analysis model was a marginal risk ratio model for the binary mortality outcome, with fixed effects for treatment (PPI, H2RB) and period (1, 2) and a logarithmic link function. Estimation was via GEE with a working exchangeable correlation matrix for within-ICU clustering, together with robust standard errors in the event the true correlation was not exchangeable. Results were expressed as a risk ratio with 95 % CI; supplementary analyses used a generalized linear mixed model with a binary outcome, the same fixed effects, and random effects for ICU and ICU-period, producing results to within 0.01 of that from the GEE analysis.

## Special considerations

### Small sample size

When the number of clusters is small (typically <30), both the GLMM and GEE approaches may lead to inflated type I error rates and require appropriate corrections [16]. These corrections involve a modification of the number of degrees of freedom used to construct confidence intervals and perform statistical tests but may also involve a correction to the estimates of the standard errors them-selves [17]. While these corrections maintain a desirable type I error rate, their use is associated with a loss in power that needs to be accounted for in the sample size calculation. Although not as flexible, cluster-level analyses are particularly suitable when the number of clusters is very small and do not require the use of corrections. Furthermore, the risk of baseline imbalance increases when only a few clusters are randomized [18]. This may introduce bias in the intervention effect estimate if unbalanced variables are strongly associated with the outcome. Both GLMM and GEE approaches allow for adjustment on baseline covariates; however, the adjustment strategy must be pre-specified in the statistical analysis plan [19].

### Analysis of cohort trials

Our example trials all involve cross-sectional data collection meaning few, if any, individuals are measured more than once in the trial. The models we present for each design also assume cross-sectional data collection. Where the same individuals are measured (provide outcome values) repeatedly throughout the trial (a cohort trial) a simple extension to the mixed models we have presented is to add a random effect (intercept) for each individual. In combination with the random effect for cluster, this specifies what is termed a ‘nested exchangeable’ correlation structure. More complex correlation structures can be specified ‘within individual’ just as ‘within cluster’, for either ‘closed’ cohort trials or ‘open’ cohort trials (where individuals leave and join clusters during the trial, e.g. care homes) as explained for the analysis of stepped wedge trials in Li et al. [12].

### Missing data

As in other settings, the choice of the method to handle the missing data depends on the missingness mechanism. If data are missing completely at random, that is, the probability of the outcome being missing does not depend on any observed or unobserved variable, a complete case analysis, excluding any participant with a missing outcome can provide an unbiased estimate of the intervention effect, but will lead to a reduction in power. A complete case analysis may also be valid (but underpowered) when missingness depends on observed covariates, as long as these covariates are included in the analysis model [20]. If data are missing at random, that is, the probability of missing outcome is independent of unobserved data conditionally on observed data, multiple imputation can be considered; however, the imputation model must capture the clustered nature of the data, using multilevel multiple imputation [21] and be consistent with the analysis model .e.g. if GLMM is used to analyze the data, GLMM must be used to impute the data [22]. Finally, if data are missing not at random, the probability of missing outcome depending on unobserved variables, multiple imputation is not appropriate, but sensitivity analyses are recommended to assess the robustness of the results [23]. In some instances, an entire cluster may drop out during the conduct of a cluster randomized trial. For more discussion on this issue see [24].

### ICC estimation

Along with the intervention effect estimate and its confidence interval, the ICC estimate and corresponding confidence interval should be reported when analyzing a CRT. This estimate is key in understanding the strength of clustering in the data and to gauge whether the assumed ICC for the sample size calculation was reasonable. It also helps researchers when designing future trials. For a continuous outcome, the ICC can be easily obtained from the output of the linear mixed model, as the ratio of the variance of the random effect over the total variance of the outcome (i.e. the sum of the residual variance and the between-cluster variance). For binary outcomes, the ICC depends on the outcome prevalence, and it is therefore recommended to estimate the ICC separately in each intervention arm [25]. Moreover, there are several estimators of the ICC proposed for binary outcomes [26], and while they cannot be easily calculated from the output of the logistic mixed effect model, most statistical software have built-in commands for their computation. For other types of outcomes (count or time-to-event), the ICC is not defined, but the coefficient of variation can be used instead, and is expressed as the ratio of the between-cluster variance and the average cluster mean [5]. It is important to note that the ICC estimate is often very imprecise, especially when the number of clusters is small.

### Estimands

Normally the primary analysis of a CRT will be under the ‘intention to treat’ or ‘as randomised’ principle. This pragmatic approach aims to estimate the effect of trying to implement the intervention on outcomes and is unbiased as it respects the randomisation. In longitudinal trials such as SW and CRXO, intention to treat is normally interpreted to mean analysis according to the intended exposure for the sequence at any period. Other approaches may be considered where interest is in the effect of the intervention if fully implemented. For simple, cluster-level, interventions it may be easy to identify which clusters implemented the intervention and which did not and compare the implementing clusters to control clusters in a ‘per protocol’ analysis. Such an analysis is however susceptible to bias, as implementing and non-implementing intervention arm clusters may differ in other ways, and randomisation is not respected. Alternatively, a ‘principal stratum’ analysis approach can be taken, just as has been proposed for individually randomised trials as part of the definition of the trial ‘estimand’ [27]. This method aims to estimate the average effect of the intervention in clusters that would implement it. In general, however, defining and measuring the level of implementation in cluster randomised trials may be difficult, particularly if there are multiple intervention components at cluster and individual level or in longitudinal designs where clusters may fully implement interventions, but not at the intended time.

## Summary

When analysing cluster trials, clustering must be taken into account in every analysis. This can be done using either cluster-level or individual-level analyses. In addition, for cluster trials with multiple periods such as SWT and CRXO, one should always adjust for the effect of time to avoid biases due to potential confounding.

For SW and CRXO, extensions to the basic analysis model include complex correlation structures and varying intervention effects. Other key aspects to consider include corrections in trials with a small number of clusters, handling of missing data, choice of estimands and ICC calculation. All these analytical choices must be prespecified in a detailed analysis plan. When there is uncertainty about the primary analysis method, sensitivity analyses should be considered to check the robustness of the results to different assumptions. A summary of key recommendations is provided (Table 1).

The analysis of cluster trials can be complex; however, there is a growing number of tools available including online resources and software packages [28]. Analyses should be reported in accordance with recommendations from the relevant CONSORT statements [29,30].

## Figures and Tables

**Table 1:** Key considerations

Adjust for within-cluster correlations Adjust for time trends when analyzing stepped-wedge trials Consider different correlations between periods compared to within periods in trials with multiple periods Use corrections when the number of clusters is small Select an appropriate approach for handling missing data Choose estimands based on the effect of interest Calculate and report the ICC Pre-specify analyses in a statistical analysis plan Report according to CONSORT recommendations
